# Complete chloroplast genome of *Ilex dabieshanensis*: Genome structure, comparative analyses with three traditional *Ilex* tea species, and its phylogenetic relationships within the family Aquifoliaceae

**DOI:** 10.1371/journal.pone.0268679

**Published:** 2022-05-19

**Authors:** Ting Zhou, Kun Ning, Zhenghai Mo, Fan Zhang, Yanwei Zhou, Xinran Chong, Donglin Zhang, Yousry A. El-Kassaby, Jian Bian, Hong Chen

**Affiliations:** 1 Institute of Botany, Jiangsu Province and Chinese Academy of Sciences (Nanjing Botanical Garden Mem. Sun Yat-Sen), The Jiangsu Provincial Platform for Conservation and Utilization of Agricultural Germplasm, Nanjing, China; 2 College of Horticulture, Jinling Institute of Technology, Nanjing City, Jiangsu Province, P.R. China; 3 Department of Horticulture, University of Georgia, Athens, GA, United States of America; 4 Department of Forest and Conservation Sciences, University of British Columbia, Vancouver, BC, Canada; 5 Jiangsu Yufeng Tourism Development Co. Ltd., Yancheng, China; Institute for Biological Research, University of Belgrade, SERBIA

## Abstract

*Ilex dabieshanensis* K. Yao & M. B. Deng is not only a highly valued tree species for landscaping, it is also a good material for making kuding tea due to its anti-inflammatory and lipid-lowering medicinal properties. Utilizing next-generation and long-read sequencing technologies, we assembled the whole chloroplast genome of *I*. *dabieshanensis*. The genome was 157,218 bp in length, exhibiting a typical quadripartite structure with a large single copy (LSC: 86,607 bp), a small single copy (SSC: 18,427 bp) and a pair of inverted repeat regions (IRA and IRB: each of 26,092 bp). A total of 121 predicted genes were encoded, including 113 distinctive (79 protein-coding genes, 30 tRNAs, and 4 rRNAs) and 8 duplicated (8 protein-coding genes) located in the IR regions. Overall, 132 SSRs and 43 long repeats were detected and could be used as potential molecular markers. Comparative analyses of four traditional *Ilex* tea species (*I*. *dabieshanensis*, *I*. *paraguariensis*, *I*. *latifolia* and *I*. *cornuta*) revealed seven divergent regions: *matK*-*rps16*, *trnS*-*psbZ*, *trnT*-*trnL*, *atpB*-*rbcL*, *petB*-*petD*, *rpl14*-*rpl16*, and *rpl32*-*trnL*. These variations might be applicable for distinguishing different species within the genus *Ilex*. Phylogenetic reconstruction strongly suggested that *I*. *dabieshanensis* formed a sister clade to *I*. *cornuta* and also showed a close relationship to *I*. *latifolia*. The generated chloroplast genome information in our study is significant for *Ilex* tea germplasm identification, phylogeny and genetic improvement.

## Introduction

In green plants, the chloroplast is a photosynthetic organelle with fundamental roles in carbon fixation and energy production [1, 2]. It possesses its own independent genome featuring maternal, paternal, or biparental inheritance and a relatively conserved four-part circular structure consisting of a large single copy (LSC), a small single copy (SSC), and two inverted repeat regions (IRs) [3–5]. Generally, land plant chloroplast genomes range in size from 120 to 160 kb, encoding 110–130 distinct genes [6, 7]. Due to their small size, reduced recombination, slow evolutionary rate, and mostly maternal transmission, chloroplast genomes of angiosperms have been extensively utilized for species identification, biodiversity evaluation, phylogenetic analysis, origin judgment, and explorations of the genetic basis for climatic adaptation [8–11]. Recently, sequencing plant chloroplast genomes has become much easier with the rapid development of sequencing technologies, such as the Illumina and PacBio sequencing platforms.

The genus *Ilex* L. (holly), Aquifoliaceae, is the largest angiosperm woody dioecious genus, cultivated as ornamental, culinary, and pharmaceutical plants [12]. Approximately 600 *Ilex* species are distributed worldwide from tropical to temperate regions, including China where more than 200 species have been documented [13, 14]. Although the taxonomy of the genus *Ilex* had been proposed based on biogeography and morphology, conducting species identifications and understanding their evolutionary relationships within and among these putative clades was complicated due to apparent morphological similarities, interspecific hybridization, and genetic introgression [15–17]. At least 20 *Ilex* species chloroplast genomes have already been released in the GenBank database. However, genomic information on many high-value species in the family Aquifoliaceae requires further investigation.

*Ilex dabieshanensis* K. Yao & M. B. Deng (Yao and Deng, 1987) is not only a highly valued tree species for landscaping, it is also a good material for making Kuding tea due to its anti-inflammatory and lipid-lowering properties. To better understand its genetic information, we sequenced and assembled the complete chloroplast genome of *I*. *dabieshanensis* (MW292560) using Illumina and PacBio sequencing technologies. Then, we carried out comparative analyses between the resulting *I*. *dabieshanensis* chloroplast genome and previously published genomes of *I cornuta* (MK335536), *I*. *latifolia* (KX426465), and *I*. *paraguariensis* (KP016928). The three species were selected because they are pharmaceutical plants used as Kuding tea, like *I*. *dabieshanensis* [18]. Our objectives were: 1) exploring the molecular structure of *I*. *dabieshanensis* chloroplast genome; 2) examining possible simple sequence and long repeats; 3) discovering hypervariable regions that could be used as specific DNA markers for the genus *Ilex*; 4) revealing the phylogenetic relationships of *I*. *dabieshanensis* within the family Aquifoliaceae; and 5) providing molecular data for *Ilex* tea germplasm identification, phylogeny, and genetic improvement.

## Materials and methods

### Plant material, chloroplast DNA extraction and sequencing

Fresh leaves of *I*. *dabieshanensis* were collected from one plant in Nanjing Botanical Garden, Jiangsu Province, China, (32°03′N latitude, 118°49′E longitude) and immediately flash-frozen in liquid nitrogen. Total chloroplast DNA was extracted using the improved sucrose gradient centrifugation method [19]. DNA integrity, purity, and concentration were estimated by 1% agarose-gel electrophoresis, a NanoDrop spectrophotometer (Thermo Scientific, Waltham, MA, USA), and a Qubit fluorometer (Life Technologies, Darmstadt, Germany), respectively. Only samples with good purity (OD _260/280_≥1.8, OD _260/230_≥1.8) were retained for sequencing. After DNA detection, two libraries with insert sizes of 300 bp and 10 kb were constructed. Chloroplast genome sequencing of the two *I*. *dabieshanensis* libraries was then performed on an Illumina HiSeq X Ten instrument (Biozeron, Shanghai, China) and a PacBio Sequel platform (Biozeron, Shanghai, China), respectively. The qualities of the resulting Illumina and PacBio raw data were filtered by FastQC.

### Chloroplast genome assembly and annotation

Using SOAPdenova software (v2.04), clean Illumina reads were first assembled with the default parameters into principal contigs [20]. Then, all contigs were classified and connected into a single draft sequence using the Geneious (v11.0.4) [21]. The BLASR software was immediately used to compare the PacBio clean data with the single draft sequence and meanwhile to extract the correction and error correction [22]. Next, scaffolds were generated by assembling the corrected PacBio clean data with the default parameters using Celera Assembler (v8.0) [23]. For gap closure, these assembled scaffolds were mapped back to the Illumina clean reads using GapCloser (v1.12) [20]. Finally, the assembled chloroplast genome of *I*. *dabieshanensis* was produced.

The chloroplast genome genes, including the predicted protein-coding gens, transfer RNA (tRNA) gens, and ribosomal RNA (rRNA) genes, were annotated using the online tool DOGMA (Dual Organellar Genome Annotator) with the default parameters and manually checked [24]. BLASTn searches against the National Center for Biotechnology Information (NCBI) website and the tRNAscanSE program were further used to determine and confirm both tRNA and rRNA genes [25]. With the default parameters and subsequent manual editing, the circular map of *I*. *dabieshanensis* chloroplast genome was drawn using OGDRAWv1.3.1 [26].

### *I*. *dabieshanensis* SSRs, long repeats, and codon usage analyses

*Ilex dabieshanensis* simple sequence repeats (SSRs) were identified by MIcroSAtellite (MISA) (http://pgrc.ipk-gatersleben.de/misa/) with the parameters set as follows: 8 for mono-, 5 for di-, 4 for tri-, and 3 for tetra-, penta-, and hexa-nucleotides [27]. Long repeats, including forward, complement, reverse, and palindromic, were analyzed using the online software REPuter with a minimum repeat size of 30 bp and a hamming distance of 3 [28]. Codon usage analysis was performed using a critical parameter of Relative Synonymous Codon Usage (RSCU) calculated by MEGA7 software [29].

### Sequence divergence analyses of the four *Ilex* species

Taking the annotation of *I*. *dabieshanensis* as the reference, we compared its complete chloroplast genome with three other *Ilex* species in the family Aquifoliaceae (*I*. *cornuta*, *I*. *latifolia*, and *I*. *paraguariensis*) using the mVISTA program (http://genome.lbl.gov/vista/mvista/submit.shtml) in Shuffle-LAGAN mode [30]. Variations in LSC/IRB/SSC/IRA region borders were also compared using IRscope (https://irscope.shinyapps.io/irapp/) [31].

### The genus *Ilex* and family Aquifoliaceae phylogeny

To determine the phylogenetic positions of *I*. *dabieshanensis* in the genus *Ilex* and family Aquifoliaceae, 21 plastid genome sequences (including 19 *Ilex* species) were retrieved from the NCBI GenBank database. Setting *Helwingia chinensis* and *H*. *himalaica* as outgroups, a phylogenetic tree was constructed in MEGAX using the maximum likelihood (ML) method based on the Tamura–Nei nucleotide substitution model [32]. The analysis was performed with 1,000 bootstrap replicates.

## Results and discussion

### *I*. *dabieshanensis* chloroplast genome features

**Fig 1:**
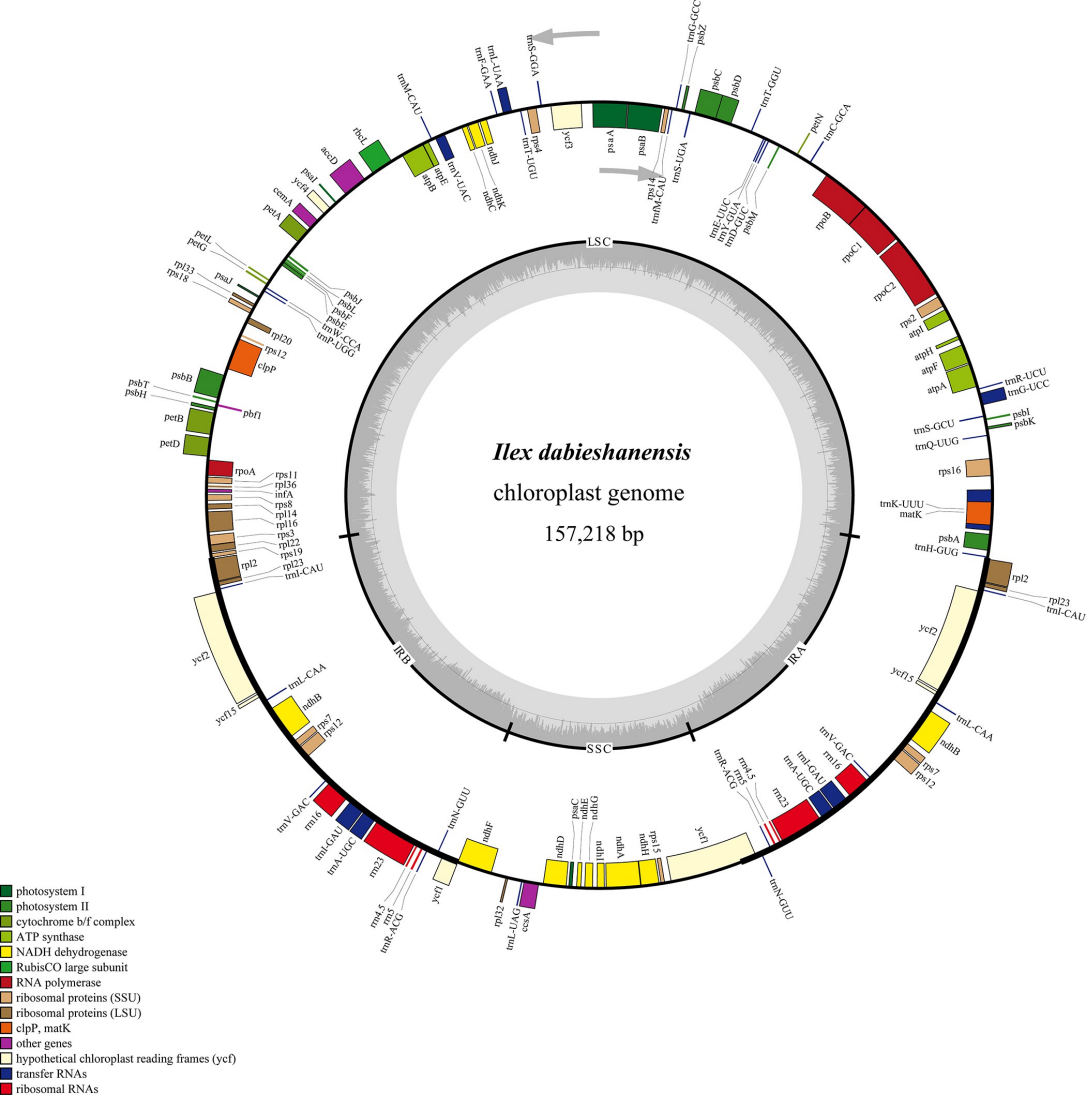
Circular gene map of *I*. *dabieshanensis* chloroplast genome. The gray arrowheads indicate gene directions. Genes inside and outside the circle represent transcribed clockwisely and counterclockwisely. Different functional genes are color coded. The innermost darker gray conforms to GC content, whereas the lighter gray conforms to AT content. LSC, large single copy region; SSC, small single copy region; IR, inverted repeat.

The complete circular chloroplast genome of *I*. *dabieshanensis* (157,218 bp in length) exhibited a typical quadripartite structure, consisting of a LSC (86,607 bp), a SSC (18,427 bp), and a pair of IR (IRA and IRB: each of 26,092 bp) regions (Fig 1 and Table 1). Like other Aquifoliaceae chloroplast genomes, it had a low GC content (37.69%) [33]. The IR regions had higher GC contents (42.94%) than the LSC (35.75%) and SSC (31.93%) regions, which is a common phenomenon among plant chloroplast genomes [34–36]. The relatively high GC content of the IR regions was mostly attributed to rRNA and tRNA genes as they occupied a greater area than the protein-coding genes [37].

**Table 1:** Summary of *I*. *dabieshanensis* chloroplast genome.

Genome Features	*I*. *dabieshanensis*
Genome size (bp) / GC content (%)	157,218 / 37.69
LSC size (bp) / GC content (%)	86,607 / 35.75
SSC size (bp) / GC content (%)	18,427 / 31.93
IR size (bp) / GC content (%)	26,092 / 42.94
Total gene number	121
Unique gene number	113
Protein-coding gene	79
tRNAs	30
rRNAs	4
Genes duplicated in IRs	8

LSC, large single copy region; SSC, small single copy region; IR, inverted repeat.

A total of 121 predicted genes, including 113 unique (79 protein-coding genes, 30 tRNAs, and 4 rRNAs) and 8 duplicated (8 protein-coding genes) located in the IR regions, were identified in *I*. *dabieshanensis* chloroplast genome (Tables 1, 2 and S1 Table). Common to many plants [38, 39], the *I*. *dabieshanensis* chloroplast genome had 18 intron-containing genes—6 tRNA and 12 protein-coding genes. Among these, 16 genes contained a single intron and 2 genes, *clpP* and *ycf3*, contained two introns (Table 2). Notably, the intron of the *trnK-UUU* gene, which contains the *matK* gene, was the longest, reaching up to 2,559 bp. The *rps12* gene was a trans-spliced gene with one exon located in the LSC region (5’ end) and the other two exons (separated by an intron) located in both IRs. These findings are in line with those of the *Zingiber*, *Cymbidium*, and *Forsythia* chloroplast genomes [40–42].

**Table 2:** List of genes in the chloroplast genome of *I*. *dabieshanensis*.

Category	Group of Genes/Function	Name of Genes
Photosynthesis	Subunits_of_photosystem_I	*psaA*, *psaB*, *psaC*, *psaI*, *psaJ*
	Subunits_of_photosystem_II	*psbA*, *psbB*, *psbC*, *psbD*, *psbE*, *psbF*, *psbH*, *psbI*, *psbJ*, *psbK*, *psbL*, *psbM*, *psbT*, *psbZ*
	Subunits_of_NADH_dehydrogenase	*ndhA*^*b*^, *ndhB*^*a*^^*b*^, *ndhC*, *ndhD*, *ndhE*, *ndhF*, *ndhG*, *ndhH*, *ndhI*, *ndhJ*, *ndhK*
	Subunits_of_cytochrome_b/f_complex	*petA*, *petB*^*b*^, *petD*^*b*^, *petG*, *petL*, *petN*
	Subunits_of_ATP_synthase	*atpA*, *atpB*, *atpE*, *atpF*^*b*^, *atpH*, *atpI*
	Large_subunit_of_Rubisco	*rbcL*
Self-replication	Large_subunits_of_ribosome	*rpl14*, *rpl16*^*b*^, *rpl2*^*a*^^*b*^, *rpl20*, *rpl22*, *rpl23*^*a*^, *rpl32*, *rpl33*, *rpl36*
	Small_subunits_of_ribosome	*rps11*, *rps12*^*a*^^*b*^^*c*^, *rps14*, *rps15*, *rps16*^*b*^, *rps18*, *rps19*, *rps2*, *rps3*, *rps4*, *rps7*^*a*^, *rps8*
	DNA-dependent_RNA_polymerase	*rpoA*, *rpoB*, *rpoC1*^*b*^, *rpoC2*
	Ribosomal_RNAs	*rrn16*, *rrn23*, *rrn4*.*5*, *rrn5*
	Transfer_RNAs	*trnA-UGC*^*b*^^*b*^, *trnC-GCA*, *trnD-GUC*, *trnE-UUC*, *trnF-GAA*, *trnG-GCC*, *trnG-UCC*^*b*^, *trnH-GUG*, *trnI-CAU*, *trnI-GAU*^*b*^, *trnK-UUU*^*b*^, *trnL-CAA*, *trnL-UAA*^*b*^, *trnL-UAG*, *trnM-CAU*, *trnN-GUU*, *trnP-UGG*, *trnQ-UUG*, *trnR-ACG*, *trnR-UCU*, *trnS-GCU*, *trnS-GGA*, *trnS-UGA*, *trnT-GGU*, *trnT-UGU*, *trnV-GAC*, *trnV-UAC*^*b*^, *trnW-CCA*, *trnY-GUA*, *trnfM-CAU*
Other genes	Maturase	*matK*
	Protease	*clpP* ^*b*^
	Envelope_membrane_protein	*cemA*
	Acetyl-CoA_carboxylase	*accD*
	C-type_cytochrome_synthesis_gene	*ccsA*
	Translation_initiation_factor	*infA*
Unknown genes	Proteins_of_unknown_function	*ycf1*^*a*^, *ycf15*^*a*^, *ycf2*^*a*^, *ycf3*^*b*^, *ycf4*

^*a*^ Two gene copies in IR_S_

^b^ Genes containing introns

^c^ Genes divided into two independent transcription units.

### SSR and long repeats identification

**Fig 2:**
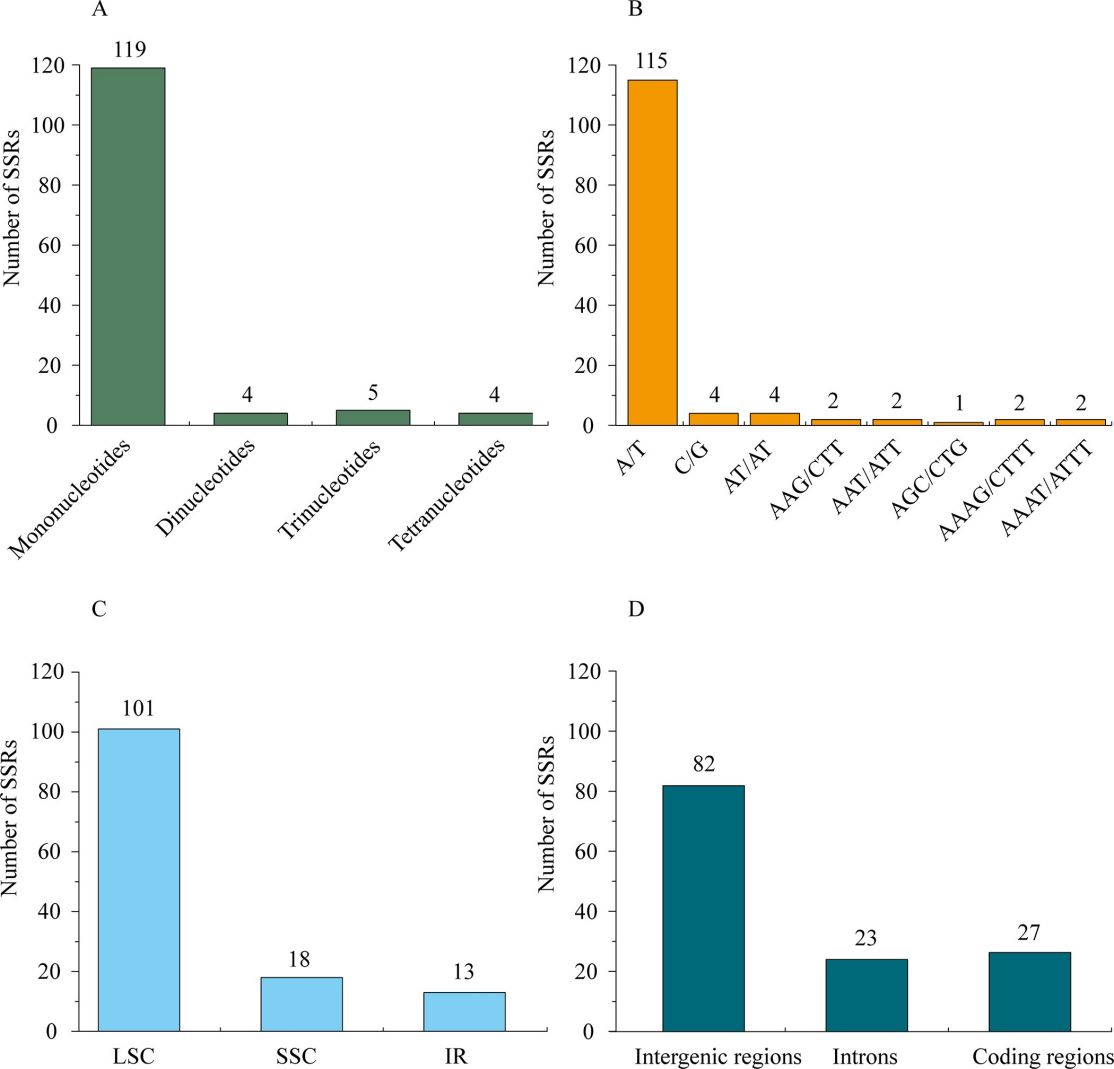
Distribution of SSR Types in *I*. *dabieshanensis* chloroplast genome. A) Number of SSRs detected; B) Number of identified SSRs in different repeat class types; C) Number of identified SSRs in different genomic regions; and D) Number of identified SSRs in intergenic regions, introns, and coding regions.

Short (1–6 nucleotides) and long (10–100 nucleotides) repeats are distinguished according to the number of nucleotides in the repeat units [43]. SSRs (simple sequence repeats), or microsatellites, are short tandem repeats. Generally, SSRs have been widely used in population genetic and phylogenetic studies for their extensive distribution throughout the chloroplast genome and significant effects on its recombination and rearrangement [44–46]. In *I*. *dabieshanensis* chloroplast genome, 132 SSRs were determined by MISA, among which, there were 119 mononucleotides, 4 dinucleotides, 5 trinucleotides, and 4 tetranucleotides, with a size of at least 8 bp (Fig 2A and S2 Table). The majority of SSRs were mono- and di-nucleotides, which was supported by previously reported chloroplast genome information [47, 48]. Among these nucleotides, A/T mononucleotides were the most abundant (accounting for 87.12%), similar to reports that short polyadenine (polyA) or polythymine (polyT) repeats are the main types of SSRs in chloroplast genomes (Fig 2B) [49]. Different regions possessed different numbers of SSRs. As shown in Fig 2C, there were 101, 18, and 13 SSRs located in the LSC, SSC, and IR regions, respectively. Shown in Fig 2D, 82, 23, and 27 SSRs were separately detected in intergenic regions, introns, and coding regions. These results corresponded with earlier reports that SSRs identified in *Ilex* chloroplast genome were primarily located in the LSC regions. SSRs were also enriched in noncoding regions [33, 48]. The SSR primers excavated in *I*. *dabieshanensis* chloroplast genome could be applied to future *Ilex* population genetic studies, polymorphism investigations, and evolution analyses.

**Fig 3:**
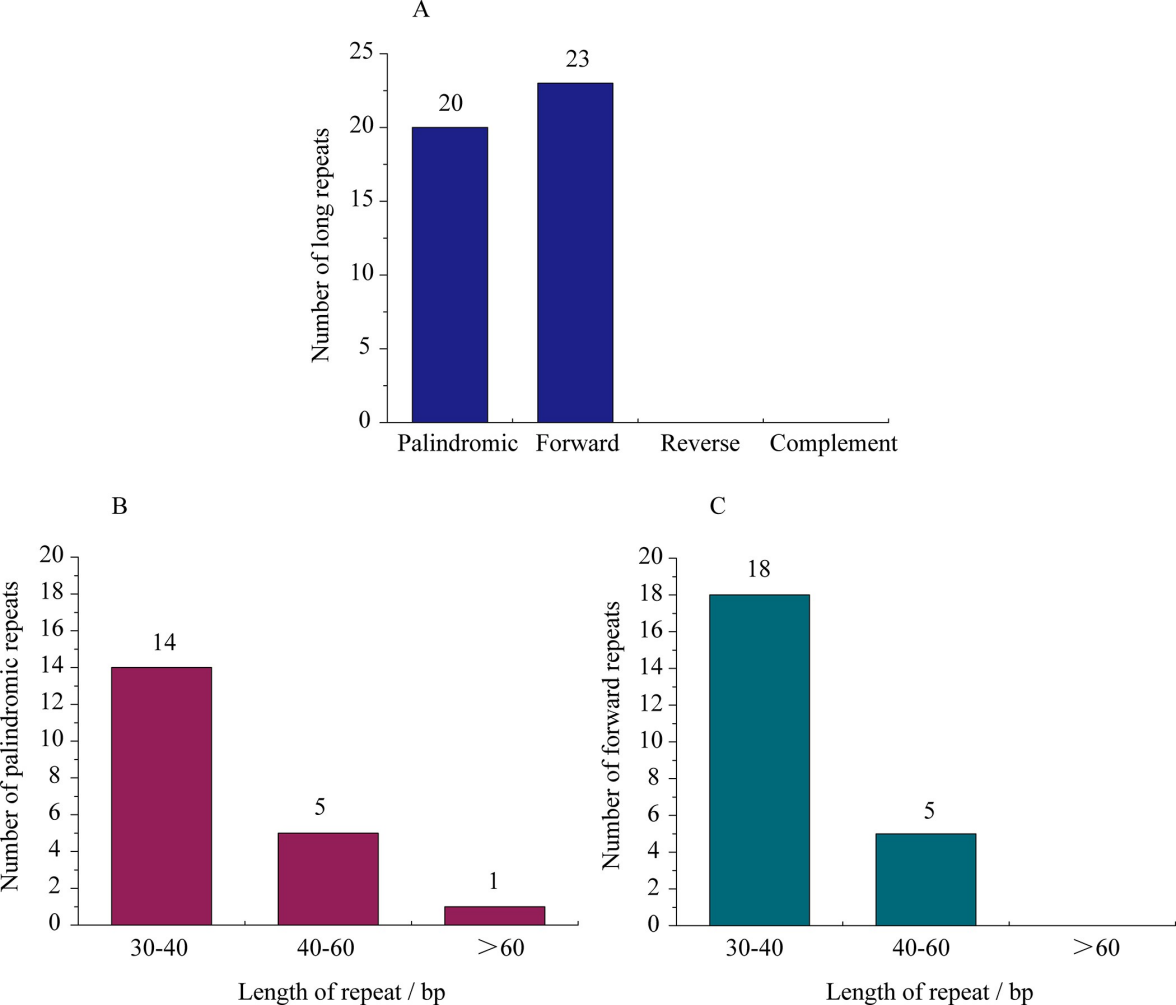
Analysis of identified long repeat sequences in *I*. *dabieshanensis* chloroplast genome. A) Number of long repeats in four identified types; B) Number of palindromic repeats by length; and C) Number of forward repeats by length.

For the majority of land plants, long repeats in the chloroplast genome are considered uncommon [38]. In terms of direction and complementarity, these repeats can be divided into four types: forward, reverse, complement, and palindromic [50]. Altogether, 43 long repeats (20 palindromic and 23 forward) were identified in *I*. *dabieshanensis* chloroplast genome and 30–40 bp repeats were the most frequent, similar to several other land plants [51, 52]. No reverse or complementary repeats were identified in *I*. *dabieshanensis* chloroplast genome, same in *I*. *latifolia* chloroplast genome (Fig 3 and S3 Table) [33].

### Codon preference analysis

Codon usage patterns and nucleotide composition can provide important information on the genetic modification of land plant chloroplast genomes. In general, no bias occurred in codon usage, or synonymous codon usage, when selective pressure was absent [53]. However, during plant evolution, the pattern of synonymous codon usage usually exhibited preference [54]. Relative synonymous codon usage (RSCU) is used as an effective index to determine codon preference [55]. A RSCU≤1.0 indicates no preference, 1.0<RSCU<1.2 signifies a weak preference, 1.2≤RSCU≤1.3 represents a moderate preference, and RSCU>1.3 indicates a strong preference [53]. In *I*. *dabieshanensis* chloroplast genome, 64 types of codons (26,932 codons totally) encoded 20 different amino acids. 30 of which had RSCU>1: 1 weak preference (RSCU = 1.16), 9 moderate preference (1.21 ≤ RSCU ≤ 1.28), and 20 strong preference (1.39 ≤ RSCU ≤ 1.85) (Table 3). Interestingly, except for TTG (G-ending), the other preferred codons all ended with the base A or T, which was previously confirmed to be the norm in most chloroplast genomes [56].

**Table 3:** Codon usage in *I*. *dabieshanensis*.

Amino Acids	Codon	No.	RSCU	Amino Acids	Codon	No.	RSCU
Ala	GCA	426	1.2136	Pro	CCA	326	1.156
Ala	GCC	212	0.6039	Pro	CCC	209	0.7411
Ala	GCG	125	0.3561	Pro	CCG	156	0.5531
Ala	GCT	641	1.8262	Pro	CCT	437	1.5496
Cys	TGC	69	0.4584	Glu	CAA	734	1.5244
Cys	TGT	232	1.5415	Glu	CAG	229	0.4755
Asp	GAC	209	0.3745	Arg	AGA	505	1.8486
Asp	GAT	907	1.6254	Arg	AGG	163	0.5967
Glu	GAA	1051	1.5155	Arg	CGA	380	1.391
Glu	GAG	336	0.4844	Arg	CGC	107	0.3917
Phe	TTC	543	0.7249	Arg	CGG	135	0.4942
Phe	TTT	955	1.275	Arg	CGT	349	1.2776
Gly	GGA	759	1.6517	Ser	AGC	113	0.3242
Gly	GGC	180	0.3917	Ser	AGT	422	1.2109
Gly	GGG	320	0.6964	Ser	TCA	424	1.2166
Gly	GGT	579	1.26	Ser	TCC	334	0.9583
His	CAC	150	0.4643	Ser	TCG	187	0.5365
His	CAT	496	1.5356	Ser	TCT	611	1.7532
Ile	ATA	705	0.9288	STOP	TAA	43	1.4659
Ile	ATC	464	0.6113	STOP	TAG	24	0.8181
Ile	ATT	1108	1.4598	STOP	TGA	21	0.7159
Lys	AAA	1057	1.4783	Thr	ACA	434	1.2534
Lys	AAG	373	0.5216	Thr	ACC	248	0.7162
Leu	CTA	389	0.8244	Thr	ACG	142	0.4101
Leu	CTC	201	0.4259	Thr	ACT	561	1.6202
Leu	CTG	190	0.4026	Val	GTA	539	1.4706
Leu	CTT	602	1.2758	Val	GTC	203	0.5538
Leu	TTA	869	1.8417	Val	GTG	209	0.5702
Leu	TTG	580	1.2292	Val	GTT	515	1.4051
Met	ATG	647	1	Trp	TGG	476	1
Asn	AAC	303	0.459	Tyr	TAC	194	0.3876
Asn	AAT	1017	1.5409	Tyr	TAT	807	1.6123

### Comparative chloroplast genome analysis

**Fig 4:**
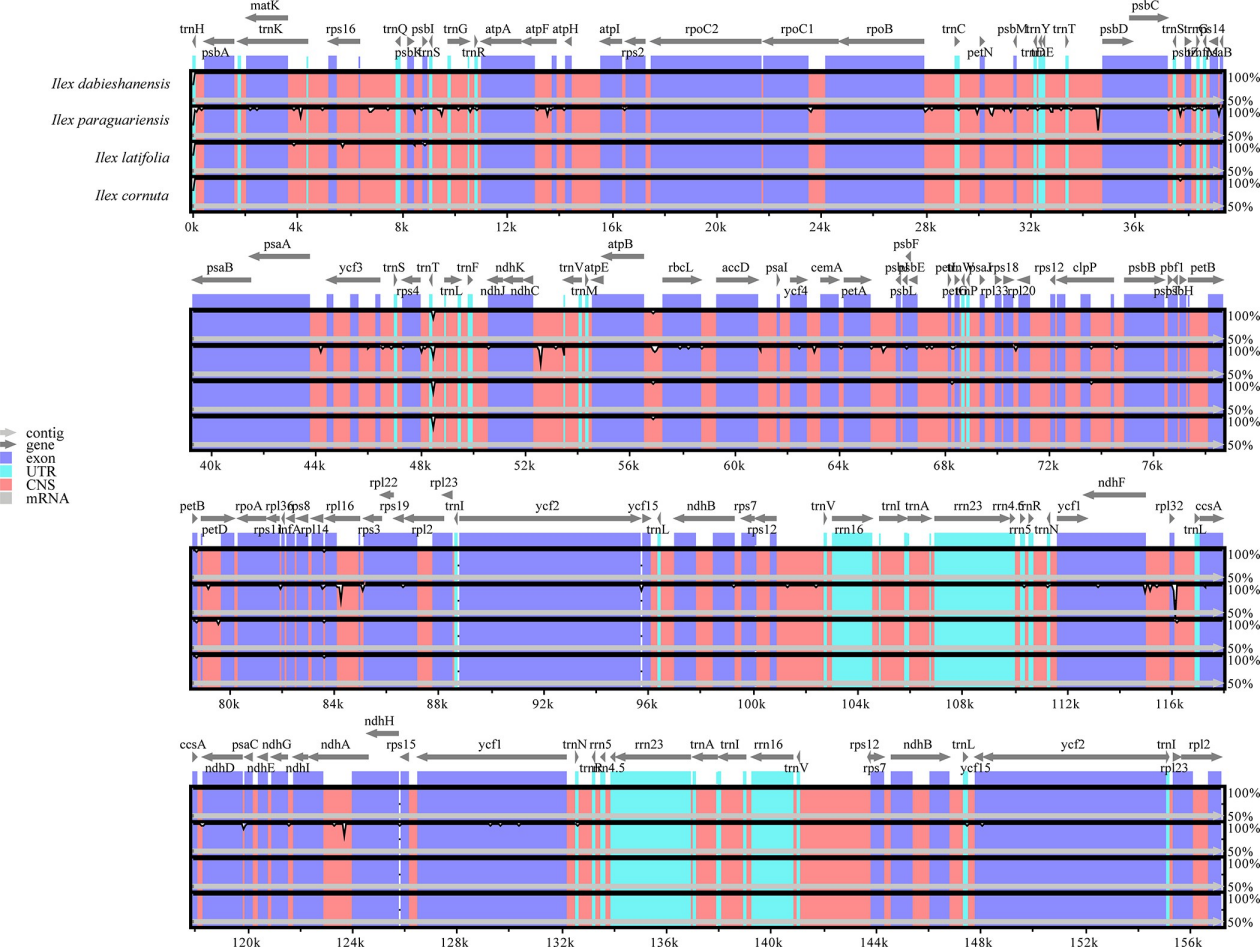
Chloroplast genome comparisons among four *Ilex* species by mVISTA. The chloroplast genome of *I*. *dabieshanensis* was used as a reference. Gray arrows above the alignment represent gene orientation. Different genome regions are color-coded as exons (purple bars), rRNA or tRNA (sky-blue bars), and non-coding sequences (CNS, pink bars). The horizontal axis indicates the coordinates within the chloroplast genome and vertical scale indicates the percentage of identity ranging from 50 to 100%. Regions with sequence variation among the four *Ilex* species were marked with white peaks.

The entire chloroplast genomes of four sequenced / published *Ilex* species were compared, with *I*. *dabieshanensis* used as the reference. Regions with sequence variation among the four *Ilex* species were marked with white peaks. Overall, the mVISTA results revealed high degrees of sequence identity between them, especially in *I*. *dabieshanensis and I*. *cornuta*, suggesting a greatly conserved evolution model. The relatively divergent regions primarily occurred in the non-coding regions rather than in coding regions, which was also previously demonstrated in the families Juglandaceae, Zingiberaceae, and Fabacaeae [8, 40, 57]. In total, seven variable sites, including *matK*-*rps16*, *trnS*-*psbZ*, *trnT*-*trnL*, *atpB*-*rbcL*, *petB*-*petD*, *rpl14*-*rpl16*, and *rpl32*-*trnL*, were subsequently detected, some of which were validated in Yao et al. (2016) [16] and Cascales et al. (2017) [58]. These variations may promote the generation of potential DNA markers for *Ilex* species identification and phylogenetic reconstruction (Fig 4).

### IR contraction and expansion

**Fig 5:**
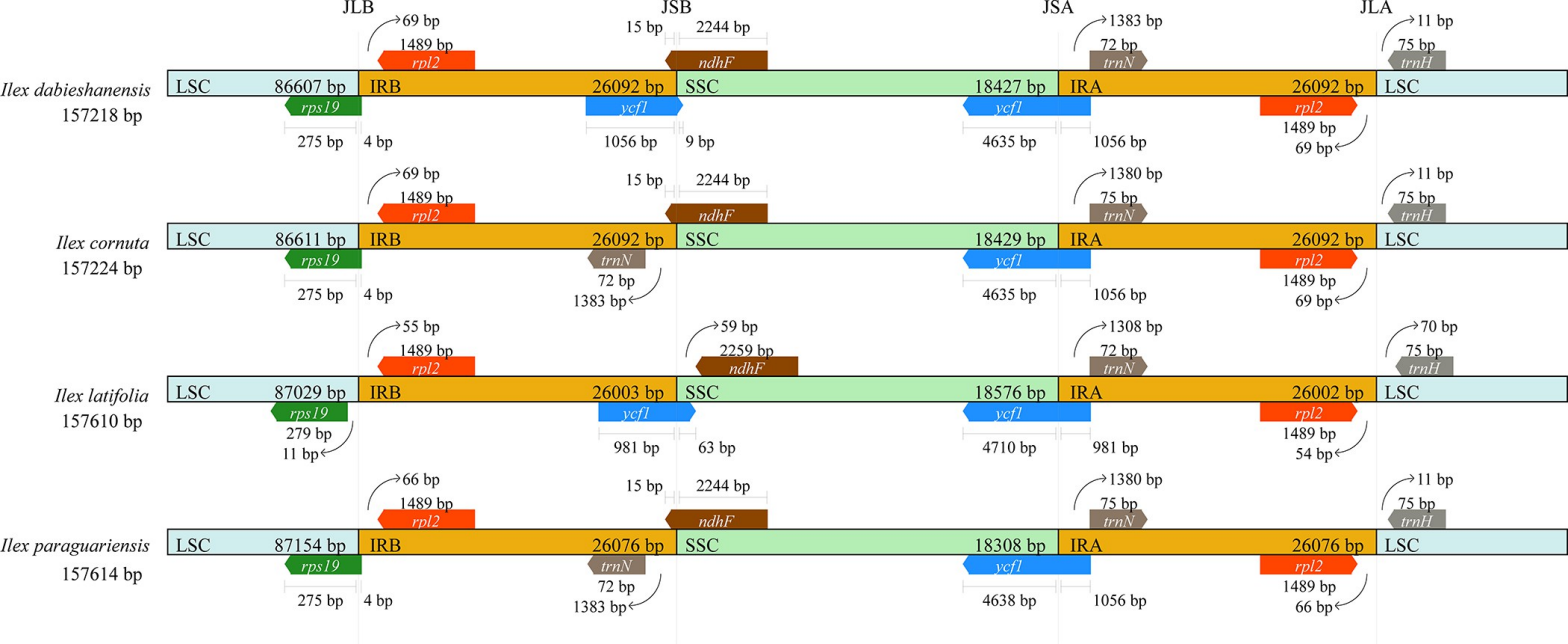
Comparisions of the SSC/IRs and LSC/IRs junctions among four *Ilex* chloroplast genomes. Colored boxes above the strip with scaled sequence length represent the denoted genes. Between boxed genes and boundaries are the gaps in base length (bp). JLB, JSB, JSA, and JLA indicate IRB/LSC, IRB/SSC, IRA/SSC, and IRA/LSC junctions, respectively.

Variations (contraction or expansion) in the IR/SC boundary regions are common, which could give rise to differences in gene size and content among chloroplast genomes, even though the IR regions were often highly conserved [59, 60]. A comparison of IR/SC junctions among the four *Ilex* chloroplast genomes (*I*. *dabieshanensis*, *I*. *cornuta*, *I*. *latifolia*, and *I*. *paraguariensis*) is presented in Fig 5. Overall, JLB (LSC/IRB), JLA (LSC/IRA), and JSA (SSC/IRA) junctions were relatively conserved, whereas JSB (SSC/IRB) junctions were strikingly different. The genes located at the JSA/B and JLA/B junctions included *rps19*, *rpl2*, *ycf1*, *ndhF*, *trnN*, and *trnH*. At the JLA and JLB boundaries, the *rpl2* gene of all four *Ilex* species was entirely located in the IRA and IRB regions, presenting a complete expansion in their IR regions. Except for *I*. *latifolia*, the *rps19* gene in *I*. *dabieshanensis*, *I*. *cornuta*, and *I*. *paraguariensis* slightly covered the JLB, showing just a partial expansion (4 bp) in the JLB boundary. The *ycf1* genes in all four *Ilex* species were throughout the JSA, which is common in angiosperms whose complete copy of *ycf1* spans the IR regions [61]. However, in JSB, it was only detected in *I*. *dabieshanensis* and *I*. *latifolia*, with an expanded length of 1,056 bp and 981 bp in IRB, respectively. The *ndhF* gene exhibited the same situation as *rps19*, separately with 15 bp gaps to the JSB in *I*. *dabieshanensis*, *I*. *cornuta*, and *I*. *paraguariensis*.

### Phylogenetic analysis of *Ilex* species in the family Aquifoliaceae

**Fig 6:**
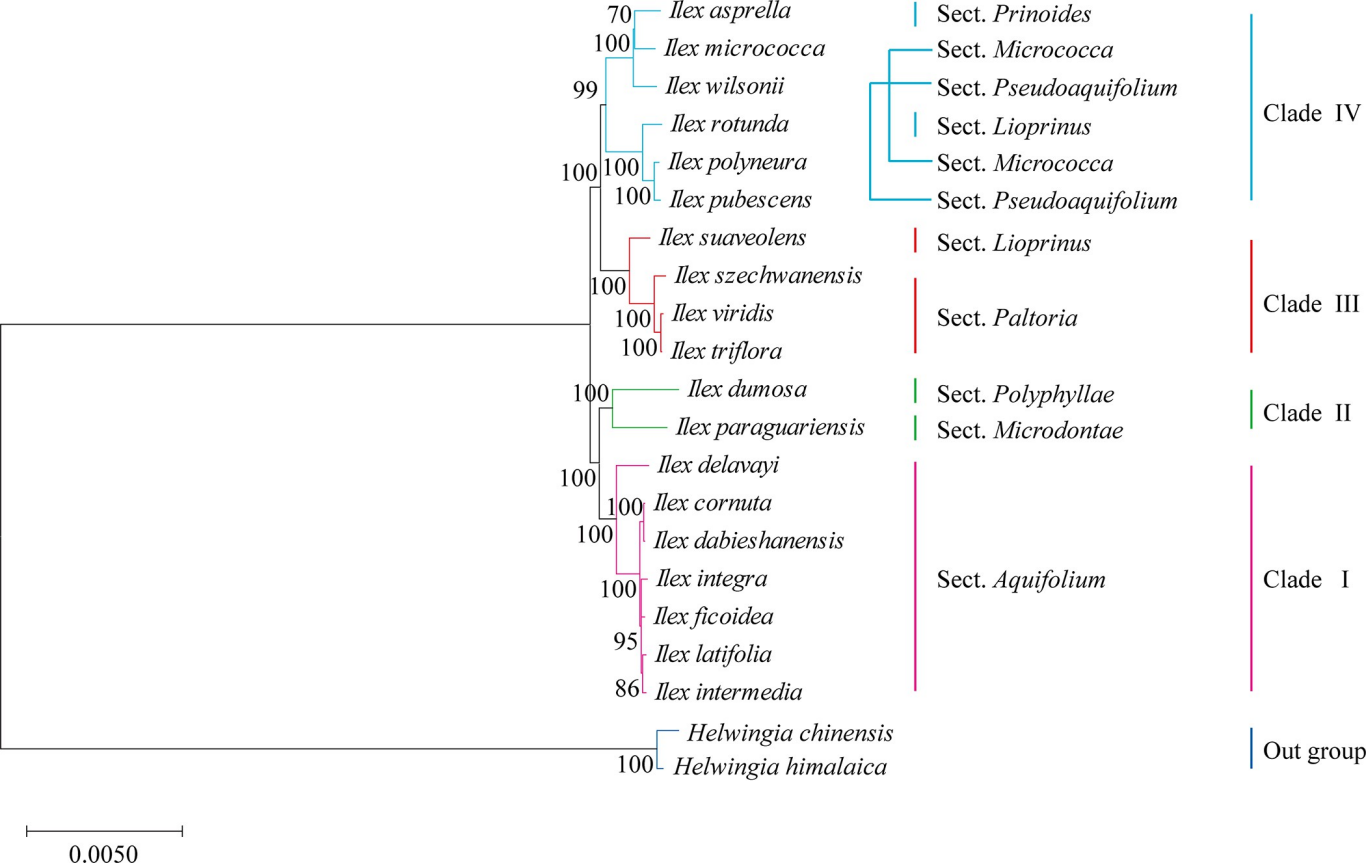
Phylogenetic tree for 19 *Ilex* species in the family Aquifoliaceae using Maximum Likelihood (ML) method, based on alignments of complete chloroplast genomes. The genomes of *Helwingia chinensis* and *H*. *himalaica* were set as outgroups. Numbers next to the branches indicated bootstrap values from 1,000 replicates.

To determine the phylogenetic relationships, the entire plastid genome sequences of 19 *Ilex* species were aligned and a phylogenetic tree was generated by MEGAX using the maximum likelihood (ML) method with 1,000 bootstrap replicates. *Helwingia chinensis* and *H*. *himalaica* were set as out groups in the analyses. As shown in Fig 6, four clades were obtained from the examined *Ilex* species. *Ilex dabieshanensis* was clustered with *I*. *cornuta* in clade I, which also included five additional *Ilex* species (*I*. *delavayi*, *I*. *integra*, *I*. *ficoidea*, *I*. *latifolia*, and *I*. *intermedia*). These species are evergreen trees with leathery leaves that are broadly distributed across subtropical Asia [33]. Our results were in accordance with Shi et al. (2016) [62], who showed that *I*. *dabieshanensis* was a natural hybrid of *I*. *cornuta* and *I*. *latifolia*. *Ilex paraguariensis* and *I*. *dumosa*, which both originated from South America, were grouped in clade II. In clade III, *I*. *trifloral*, *I*. *viridis*, *I*. *szechwanensis*, and *I*. *suaveolens* showed close relationships. Clustered in clade IV, *I*. *asprella*, *I*. *micrococca* and *I*. *polyneura* are all deciduous shrubs or trees, while the other three clades contained evergreens. Overall, the plastid phylogeny illustrated that within the family Aquifoliaceae, the *Ilex* species might originate from more than one ancestor.

## Conclusions

Here, the complete chloroplast genome of *I*. *dabieshanensis* (157,218 bp in length) was sequenced and analyzed. It was predicted to encode 121 genes, including 113 unique (79 protein-coding genes, 30 tRNAs, and 4 rRNAs) and 8 duplicated (8 protein-coding genes) located in the IR regions. A total of 132 SSRs and 43 long repeats were detected, which could be utilized as potential molecular markers. Based on the protein-coding genes, the codon usage represented a bias toward A/T-ending. Comparative chloroplast genome analyses of *I*. *dabieshanensis*, *I*. *cornuta*, *I*. *latifolia*, and *I*. *paraguariensis* revealed seven divergent regions: *matK*-*rps16*, *trnS*-*psbZ*, *trnT*-*trnL*, *atpB*-*rbcL*, *petB*-*petD*, *rpl14*-*rpl16*, and *rpl32*-*trnL*. These variations might be applicable for distinguishing different species within the genus *Ilex*. Phylogenetic reconstruction strongly suggested that *I*. *dabieshanensis* formed a sister clade to *I*. *cornuta* and also showed a close relationship to *I*. *latifolia*. The generated chloroplast genome information in our study is significant for *Ilex* tea germplasm identification, phylogeny and genetic improvement.

## Supporting information

S1 Table Genes predicted in *I*. *dabieshanensis* cp genome. (XLS)

S2 Table SSRs identified in *I*. *dabieshanensis* cp genome. (XLS)

S3 Table Long repeats in the *I*. *dabieshanensis* cp genome. (XLSX)
